# A Micro-Optic Stalk (μOS) System to Model the Collective Migration of Retinal Neuroblasts

**DOI:** 10.3390/mi11040363

**Published:** 2020-03-31

**Authors:** Stephanie Zhang, Miles Markey, Caroline D. Pena, Tadmiri Venkatesh, Maribel Vazquez

**Affiliations:** 1 Department of Biomedical Engineering, Binghamton University, 4400 Vestal Pkwy E, Binghamton, NY 13902, USA; szhan152@binghamton.edu; 2 Department of Biomedical Engineering, Rutgers University, 599 Taylor Rd, Piscataway, NJ 08854, USA; mwm104@scarletmail.rutgers.edu; 3 Department of Biomedical Engineering, City College of New York, New York City, NY 10031, USA; carolinedaniellapena@gmail.com; 4 Department of Biology, City College of New York, New York City, NY 10031, USA; tvenkatesh@ccny.cuny.edu

**Keywords:** drosophila, development, stem cells, fibroblast growth factor, chemotaxis

## Abstract

Contemporary regenerative therapies have introduced stem-like cells to replace damaged neurons in the visual system by recapitulating critical processes of eye development. The collective migration of neural stem cells is fundamental to retinogenesis and has been exceptionally well-studied using the fruit fly model of Drosophila Melanogaster. However, the migratory behavior of its retinal neuroblasts (RNBs) has been surprisingly understudied, despite being critical to retinal development in this invertebrate model. The current project developed a new microfluidic system to examine the collective migration of RNBs extracted from the developing visual system of Drosophila as a model for the collective motile processes of replacement neural stem cells. The system scales with the microstructure of the Drosophila optic stalk, which is a pre-cursor to the optic nerve, to produce signaling fields spatially comparable to in vivo RNB stimuli. Experiments used the micro-optic stalk system, or μOS, to demonstrate the preferred sizing and directional migration of collective, motile RNB groups in response to changes in exogenous concentrations of fibroblast growth factor (FGF), which is a key factor in development. Our data highlight the importance of cell-to-cell contacts in enabling cell cohesion during collective RNB migration and point to the unexplored synergy of invertebrate cell study and microfluidic platforms to advance regenerative strategies.

## 1. Introduction

Visual impairment is a global health challenge that affects growing numbers of aging and mature adults each year [1,2,3]. Regenerative therapies for the damaged visual system have introduced stem-like cells to recapitulate developmental processes and initiate regeneration in different components of the eye (reviewed in [4,5,6]). The optic nerve is central to vision, and it is developmentally preceded by a microscale structure called the optic stalk, which enables axonal targeting from the brain to the developing eye [7]. This critical neural communication is facilitated by the collective and highly coordinated migration of retinal neuroblasts (RNBs), or neural stem-like progenitors, along signaling gradients of motogenic and morphogenic factors [8,9]. Collective chemotaxis, i.e., the directional migration of cells toward signaling gradients, is essential to retinal development, where RNBs migrate in response to fields of signaling molecules while maintaining cohesive and dynamic spatial relationships with one another [10,11]. Contemporary regenerative projects have sought to recapitulate these RNB behaviors via, both, in vivo transplantation [6,12] and in vitro retinal organoid models [13,14]. However, these studies have had mixed success in recapitulating and/or studying the complex inter-, intra-, and extra-cellular signaling needed for collective RNB migration [15,16].

**Figure 1:**
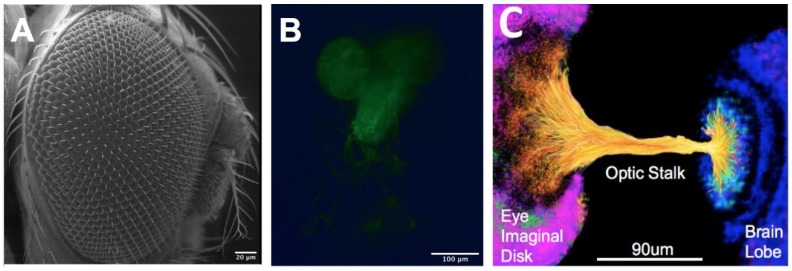
The Drosophila Melanogaster model of the developing visual system. (**A**) SEM image of the visual system of an adult fruit fly. (**B**) Representative eye–brain complex extracted from the third instar larval stage of development illustrating green fluorescent protein (GFP)+ cells of glial lineage. (**C**) Rendering of the developing optic stalk connecting the brain lobe and eye imaginal disk. Colors represent eye imaginal disc (pink), neuroblasts (yellow/orange), and brain lobe (blue).

Genetic models have illustrated that retinal development is remarkably conserved across species and has been exceptionally well-studied using Drosophila Melanogaster, or fruit fly [9,17,18]. This multi-cellular invertebrate model provides a compartmentalized system that enables direct genetic manipulation alongside in vivo observation to investigate collective processes across the optic stalk and elsewhere in the developing eye (Figure 1). Surprisingly, microdevices have been incompletely explored for the study of retinogenesis despite the established microscale of the optic stalk and wide adaptation of microsystems for organism immobilization [19,20], embryo, and larvae sorting [21,22]. However, microfluidic study of genetically manipulated cells provides a powerful and synergistic experimental model with which to examine collective RNB migration, quantitatively and mechanistically. The measurement of collective responses from defined RNBs to tunable changes in their surrounding microenvironment will greatly enrich our understanding of retinal development and advance therapeutic strategies for stem cell-based regeneration in the nervous system and elsewhere [23,24,25,26]. While numerous projects have developed chemotactic gradients to examine the migration of single cells and cell groups (reviewed in [27]), few studies have been adapted for the visual system. Contemporary projects have used microfluidics to build 3D tissue modeling for drug screening, develop bioreactors for retinal differentiation, and develop whole explant testing systems [28,29,30]. Microfluidics-based systems have also been developed to examine electrical synapses and neuromodulation in retina, as well as enable the size profiling of retinal stem cells [31,32,33]. However, very few projects have used microfluidics to examine the collective migration within developing visual systems [34,35] central to retinal regeneration therapies, and even fewer works have used invertebrate cells to do so [36,37,38,39]. Ours is the first microfluidic environment designed to represent the developing visual system of Drosophila Melanogaster to anatomical scale and used to examine collective migration therein. This device thereby provides an important testing system for studying the mechanisms of Drosophila development to advance emerging strategies for retinal regeneration.

Previous work from our group [36,37,40,41,42] has examined the collective migration of clustered retinal progenitors using microfluidic devices with characteristic lengths much larger than the physiological system examined. In this report, we describe a microfluidic device called the micro-optic stalk, or μOS, with dimensions approaching those of the developing eye–brain complex of Drosophila. Our system facilitates the study of collective RNB responses to controlled external stimuli generated within geometries that mimic spatial confinements in vivo. The results illustrate that signaling fields of the essential developmental cytokine, fibroblast growth factor (FGF), stimulate different collective chemotactic responses from RNB clusters of varying mean size. Data illustrate the preferential sizing of motile RNB groups, where smaller clusters of 3–5 cells exhibited maximum motility and directionality in response to FGF concentration gradients. Further, despite available spacing in the μOS for the migration of larger clustered RNB groups, RNBs were observed to disaggregate into smaller clusters of preferential size in response to changes in exogenous FGF concentration. These data point to the underexplored significance of cell–cell contacts in collective RNB behaviors needed to advance stem cell-based therapies [43,44] and highlight synergistic opportunities to examine these interactions, mechanistically, via concurrent genetic manipulation and controlled extracellular stimuli. 

## 2. Materials and Methods

### 2.1. Drosophila Fly Stocks

Experiments utilized the GAL4-UAS system [45], where retinal neuroblasts (RNBs) with glial markers expressed green fluorescent protein (GFP) and RNBs with neuronal markers expressed red fluorescent protein (RFP). Drosophila Melanogaster stocks of UAS-GFP (CS: Repo) and UAS-mCD8-GFP; elav GAL4 were used because the Elav (neurons) and Repo (Glia) markers are the only markers to specifically stain cells in the developing retinal ganglion [36,46]. Flies were maintained on standard corn meal agar medium and kept at 25 °C. Stocks were transferred once a week to maintain lines of larvae mixed from the two strains.

### 2.2. Dissection of Eye–Brain Complexes

Developing eye–brain complexes were extracted from third instar larvae grown from the fly stock described using established methods [47] modified by our group for cell recovery and viability [37,40]. In brief, dissection was completed in a laminar flow hood pre-treated with ultraviolet light (λ = 400 nm) (CellGard ES Energy Saver Class II, Type A2) to maintain sterility using autoclaved materials. Larvae were washed in 70% ethanol, 3X in deionized water and phosphate-buffered saline (PBS). Eye–brain complexes were carefully isolated using #5 stainless steel tweezers, and washed in 40 mL of Schneider’s medium (Thermo Fischer Scientific, Waltham, MA, USA) supplemented with 10% (vol/vol) heat-inactivated fetal bovine serum (HIFBS) and 1% (vol/vol) penicillin streptomycin. A minimum of 15 eye–brain complexes were placed in a 3-cm-diameter petri dish filled with 40 mL of PBS and placed on ice. The eye–brain complexes were further dissociated with 1 mL of 0.5 mg/mL collagenase at room temperature (25 °C) for 1 h, centrifuged at 2000 rpm for 5 min, and re-suspended in 1 mL of supplemented Schneider’s medium. The remaining tissue was mechanical pipetted in 150 μL of supplemented Schneider’s medium (10 μL per brain) and passaged through a 40-μm diameter cell strainer into a 1.5 mL conical tube to facilitate adhesion. Cell suspensions in complete Schneider’s media were maintained within a controlled 25 °C incubator (Barnstead Labline L-C incubator) with common air exchange, as is customary for Drosophila cell culture over the past two decades. This organism, and its cells, are maintained at controlled temperature without buffered carbon dioxide exchange during the life span and culture conditions [9,46,48].

### 2.3. μOS Design and Operation

**Figure 2:**
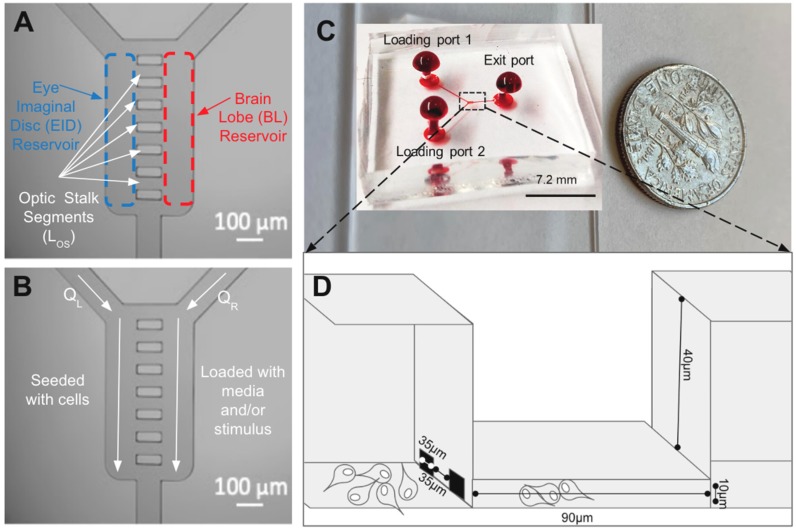
The micro-optic stalk (μOS) system is designed to represent cellular paths of retinal neuroblasts (RNBs) between the eye imaginal disk and brain lobe of the developing visual system of Drosophila Melanogaster. (**A**) Schematic of the system illustrating an array of eight horizontal microchannels (L_OS_) connected to two vertical fluidic reservoirs to represent the eye imaginal disc (EID) and brain lobe (BL). (**B**). During loading, RNBs are seeded in the EID reservoir (left) at a volume flow rate of Q_L_, while a solution of media and/or growth factor stimulus is flushed in the BL reservoir (right) at a volume flow rate of Q_R_. During testing, solutions are continually flushed in the EID and BL, while the migration or RNBs within L_OS_ segments is imaged each hour. (**C**) Image of the polydimethylsiloxane (PDMS) elastomer bonded onto a glass coverslip and with red dye to facilitate system visualization. Loading ports 1 and 2 and the Exit port are labeled alongside a dime for overall system scaling. The dashed lines indicate the region of interest. (**D**) Schematic cross-section of the μOS system after cell seeding. RNBs are seeded in the EID reservoir (left) and migrate through the OS microchannels in response to growth factor stimulus within the BL reservoir (right).

The μOS system was created to mimic the controlled microenvironment of the third instar larvae stage of the developing eye–brain complex of Drosophila, as shown in Figure 1C. The microfluidics system represents the three key components of its developing visual system: (1) the brain lobe (BL) where visual centers are developed; (2) optic stalk (OS) that enables axonal targeting from the developing brain to the visual system; and (3) eye imaginal disc (EID) where the retina is developed. Figure 2 illustrates how regions of the eye–brain complex are represented by the μOS device using fluidic reservoirs and a microchannel array. As seen, two large-volume, vertical reservoirs represent the brain lobe (BL: left) and eye imaginal disc (EID: right), which are connected by an array of eight, equally spaced microchannels. Each reservoir is 150-µm wide, 500-µm long, and 50 µm in height, to represent the large BL and EID regions. These vertical reservoirs were additionally sized to minimize the volume needed for primary cell suspensions and reduce entrance effects via a large volume ratio with the microarray (50:1) [49,50,51].

Each microchannel of the µOS array represents one OS segment and has a length (L_OS_) of 90 μm, depth of 10 μm, and characteristic width of 37 μm (W_OS1_) on the BL side and 35 μm (W_OS2_) on the EID side. We note that a height of 10 µm was selected for the L_OS_ channels to represent the cited anatomical constraints of RNB movement during this developmental stage. These dimensions are listed in Table 1, and they were chosen to most accurately represent the reported in vivo dimensions obtained via confocal imaging and dissection of this developmental stage [7,52,53]. The microchannels are spaced 35 µm apart to create multiple OS channels for concurrent RNB study to different extracellular conditions with sufficient volume for 15 brain dissections (0.016 μL) per testing condition [37]. 

**Figure 3:**
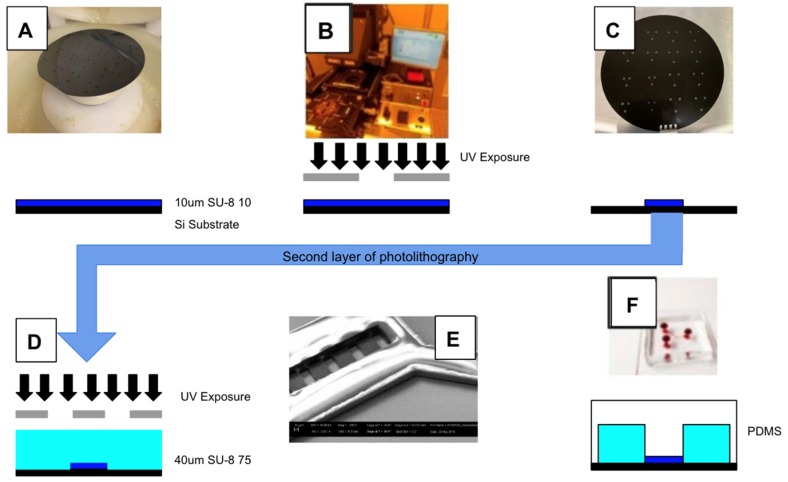
The μOS is manufactured using a two-step photolithography process with elastomeric micromolding. (**A**) The first layer of negative photoresist is spin-coated onto a silicon wafer at a height of 10 μm. (**B**) The photoresist-treated wafer is irradiated with ultraviolet light at using a mask aligner with a designed photomask. (**C**) The patterned wafer is developed and washed. (**D**) A second layer of photoresist is then applied onto the patterned wafer by repeating processes (**A**–**C**) to create a height of 50 μm for greater stability. (**E**) SEM image of a photoresist-patterned silicon wafer used for micromolding the μOS design in PDMS. (**F**) The silicon wafer undergoes silanization, enabling a mixture of polydimethylsiloxane (PDMS) to coat the wafer surface and undergo curing to create an elastomer with the desired pattern. This elastomer is peeled away from the wafer surface, cut to size, and plasma bonded onto a chemically cleaned glass slide or coverslip.

Loading of the µOS is accomplished via two inlet ports (upper left and upper right in Figure 3B) that exit the system through one outlet port directly below the array. Each loading channel is 75 µm in diameter and 3 × 10^3^ μm in length to facilitate the convective transport of molecules into the µOS geometry. The Y-shape design was selected to minimize the volume to surface area of the loading regions [55,56]. The upper left and upper right loading ports concurrently flush solutions of reagent and cell media, independently, into the vertical compartments to generate tailored concentration fields within the adjoining microchannels via bulk diffusion [55]. The BL reservoir filled with FGF is denoted as the source reservoir, and the EID reservoir filled with media is denoted as the sink reservoir in all experiments. As per Figure 2D, RNBs exit the EID reservoir and migrate toward the BL reservoir through L_OS_ segments of the µOS that represent the anatomical geometry of the developing visual system of Drosophila. Changes in the anatomical constraints experienced by cells were modeled using two-layer photolithography to produce similar differences in system height, as done previously by our group for biosystems with similar constraints [49,51].

### 2.4. System Fabrication

The μOS design was micromanufactured using a two-layer photolithography protocol and elastomeric molding previously described and used by our lab [49,57], as shown in Figure 3. In brief, a 1-mL volume of photoresist (SU-8 2010, Sigma Aldrich, St. Louis, MO, USA) was spin-coated onto a 4-inch silicon wafer (4000 rpm, 30-s) to obtain a thickness of 10μm (Figure 3A). After pre-baking (65 °C/95 °C), the wafer was irradiated (λ_UV_ = 360 nm, 180 mJ/cm^2^) using a chrome-on-glass photomask. The newly patterned wafer was then post-baked (65 °C/95 °C) on a hot plate, mechanically agitated in developer (MicroChem, Newton, MA, USA), and separately rinsed with isopropanol (IPA) and deionized water (diH2O). A second layer of photoresist (SU-8 2075, Sigma Aldrich) was spin-coated onto the patterned wafer to obtain an additional thickness of 40 μm. Then, the substrate was irradiated with UV using a mask aligner (EVG620, EV Group, Tempe, AZ, USA) to generate a system with a thickness of 50 μm on the vertical reservoirs and a 10-µm thickness for the horizontal microchannel array (Figure 3D,E). The wafer surface was silanized in a vacuum chamber for 1–2 h using a mixture of deionized water, methanol, and trichlorosilane (1H,1H,2H,H2-perfluoro-octyl). A 20-mL volume of polydimethylsiloxane (PDMS, Dow Corning, Midland, MI) was then poured onto the final patterned wafer surface and oven cured (300 °C, 15 min) to produce an elastomer of 2–3 mm in thickness. Lastly, oxygen plasma was applied to the inner surfaces of the PDMS elastomer and a piranha-cleaned glass microscope slide using a 30-s corona treatment (Electro-technic Products Inc., Chicago, IL, USA) [49,50,58] before press fitting to create the closed µOS system, as done previously by our lab [59,60] (Figure 3F).

### 2.5. Computational Transport Model and Experimental Validation

A two-dimensional numerical simulation of molecular transport within the μOS system was performed using a finite element model of the device via COMSOL Multiphysics 4.3 (COMSOL Inc., Burlington, MA). A diffusivity value of D = 1.35 × 10^−11^ mm^2^/s was used to model the in vitro transport of FGF-8 (MW = 22.4 kDa; Catalog PHG0184, Fischer Scientific), as done previously by our group and others [40,61]. Mathematical computation determined the velocity, pressure, and concentration profile within the microchannel array of the μOS system using the diffusion Equation (1) shown below.

(1) $$\frac{\partial C}{\partial t}=D\cdot \nabla^{2}C$$

where ρ is density (kg/m^3^), μ is viscosity (Pa-s), P is pressure (Pa), g is gravity (m/s^2^), D is diffusivity (m^2^/s), and C is reagent concentration (g/mol). Analysis was performed at steady state using laminar and incompressible flow (ρ = 1070 kg/m^3^) at constant viscosity (μ = 1.05 Pa-s). Boundary conditions used no slip at the inner surface walls and axial symmetry within the microchannels, while gravity effects were presumed to be negligible. Low inlet volume flow rates between 1.0 ≤ Q ≤ 5.0 μL/s were used to prevent the shearing of loaded cells [62,63], while an inlet concentration of C_o_ = 100 ng/mL of FGF was used to approach the in vivo values cited in the literature [64].

Experiments used a model fluorescent molecule of comparable molecular weight (FITC-Dextran, CAS 60842-46-8 (20 kDa), Sigma-Aldrich, St. Louis, MO) to validate the concentration distributions predicted by the computational model within the µOS. For these tests, the left inlet reservoir (L: Sink) was loaded with deionized water using a volume flow rate, Q_L_, while the right inlet reservoir (R: Source) was loaded with 100 ng/mL of dextran with a volume flow rate, Q_R_, to generate concentration gradients along the microchannel array. Several volume flow rate ratios, defined as R = Q_R_/Q_L_, were used to validate fluid flow within the μOS vertical channels and molecular transport within the horizontal microchannel array representing the optic stalk. The resulting values of fluorescence intensity, I, along the length of the horizontal microchannels, L_OS_, were measured via microscopy to estimate the changes in concentration, C, within the device.

### 2.6. Microscopy and Imaging

Scanning Electron Microscopy (Zeiss LS704U SEM, Jena, Germany) was used to capture images of the adult Drosophila compound eye at 6 kV and 2.601 A with the stage at a Z plane of 23.372 nm, as described previously by our group [36] using 20.0 kV and 2.60 A at an angle of 45°, and Z plane of 18.0 mm was used to image the patterned silicon wafer surface. A Nikon Eclipse (TE2000 Inverted, Morrell Instruments, Melville, NY, USA) was used to image cells within the µOS with and without fluorescence via NIS Elements Imaging Software and 20×/40× long working distance objectives (Nikon, Shinagawa Intercity Tower C, 2-15-3, Konan, Minato-ku, Tokyo 108-6290, Japan).

### 2.7. Device Operation and Measurement of RNB Migration

Inner surfaces of the μOS were coated with a 15 μg/mL solution of Concanavalin A (ConA) (eBioscience, Carlsbad, CA, USA) for two hours and washed with PBS under sterile conditions. This extracellular substrate was selected based on our previous work comparing RNB adhesion and viability upon a panel of substrates [37] as well as numerous citations in the Drosophila literature validating ConA as an appropriate substrate [39,48]. The coating was then aspirated, and the device washed with PBS and placed in a flow hood to air dry for 1 h. A 150-μL cell suspension was flushed into the system (NE-1000, New Era Pump Systems Inc, Farmingdale, NY, USA) at volume flow rates of Q_L_ < 5 μL/min to minimize shear [41,51] and enable RNB adhesion. Cells were left to adhere within the µOS for 2 h. Afterwards, the source reservoir (vertical compartment on the right-hand side) was continually flushed (Q_R_) with 100 ng/mL of FGF, and the sink reservoir (vertical compartment on the left-hand side) was continually flushed with cell media (Q_L_) to generate concentration gradient fields across the horizontal microchannel array.

Cell movement within the microarray was tracked as a vector in polar coordinates (radius and angle) to determine: (a) L_T_, the average total path length, which is defined as the sum of cell distances traveled over experimental times; (b) D_N_, the net displacement, which is defined as the spatial distance between the initial and final time points of motile RNBs; and (c) DR, the directionality, which is defined as the migration distance projected onto the horizontal gradient direction, using Equation (2):

(2) $$\mathrm{DR}=\sum \frac{\cos\left(\theta \right)}{n}$$

where θ is the angle between the RNB trajectory and the directional axis (horizontal) and n represents the number of RNBs [65]. Trajectories of RNBs were tracked individually using the Manual Tracking plugin from ImageJ (NIH, Bethesda, MD, USA).

### 2.8. Data Analysis and Statistics

A total of seven experiments were analyzed using three different µOS devices. Data are presented using mean ± standard error (SE) per RNB for each experiment. Live cell imaging of n = 10–15 single cells and n = 15–24 RNB clusters within the µOS microarray were obtained at 60-min intervals over an 8-h time period per experiment. One-way analysis of variance (ANOVA) for single-factor testing was used with the post-hoc Tukey test. Only values of *p* < 0.01 were considered statistically significant and reported with an asterisk, *. The maximum error was calculated using the root means square of Equation (3), where n is the number of data points.

(3) $$\mathrm{RMSE}=\sqrt{\frac{\sum {\left(y_{\mathrm{simulated}}-y_{\mathrm{real}\;\mathrm{data}}\right)}^{2}}{n}}$$

where y_simulated_ represents the simulated results from COMSOL MultiPhysics and y_real data_ represents the 20 kDa of FITC-dextran, analogous to the FGF molecular weight.

## 3. Results

### 3.1. Conserved Retinal Development

Contemporary knowledge of eye formation has been largely derived from studies of the developing visual system of Drosophila Melanogaster. Drosophila is a highly compartmentalized, multi-cellular model organism that is an ideal candidate for genetic manipulation because its highly reiterative pattern of neurons, glia, and accessory cells is readily amenable to molecular and genetic manipulations [9]. Studies on the development of the Drosophila retina have provided key insights into the signaling mechanisms underlying retinal development. Thus, the spatially and temporally orchestrated migration of RNB along the optic stalk provides a powerful microsystem to understand the cellular and molecular mechanisms that regulate collective migration.

A wealth of fly studies has demonstrated that the mechanistic processes of retinogenesis are largely conserved across species, despite stark physiological differences between the compound and mammalian eye (reviewed in [9,66]). Further, its fully mapped genome shares 60% homology with humans, enabling this invertebrate model to aid the development of medical treatments for numerous neural disorders and diseases [67]. Most significantly, the model shares comparable microscale features in its developing visual system, despite large differences in adult eye size and configuration [66,68]. Further, the integration of microfluidics with Drosophila RNBs is also advantageous because of its lower costs and absence of institutional review board (IRB) requirements associated with animal study [69]. Given these unique advantages, surprisingly few microfluidic projects have embraced fly cell study [39], and ours is among the first to develop a microscale platform of its developing visual system.

### 3.2. Validation of the μOS Chemical Environment

**Figure 4:**
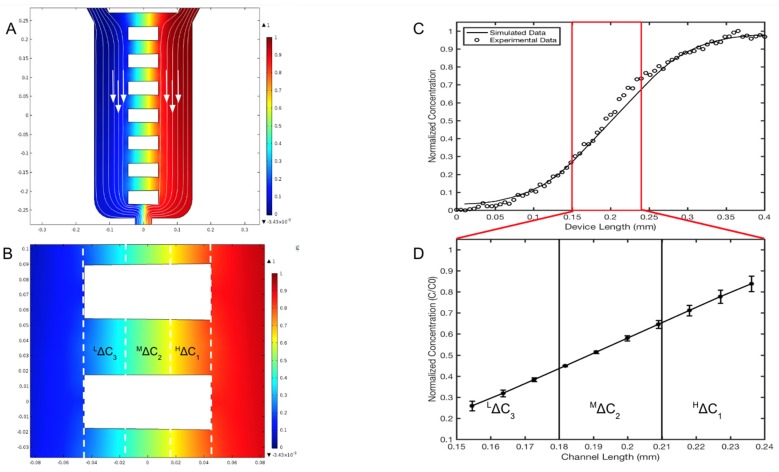
Concentration profiles generated within the μOS system are defined precisely by analytical solutions to transport processes and experimentally validated via fluorescent microscopy. (**A**) Color-coded image of computationally predicted, steady-state concentration profiles within the microarray of the μOS device. Red indicates the highest reagent concentration (C_o_) and blue indicates the minimum (C = 0). White arrows indicate the direction of the flow. (**B**) Three regions of distinct concentrations within the microarray are marked as ^H^ΔC_1_, ^M^ΔC_2_, and ^L^ΔC_3_. Three GM concentrations are established in the device of ^L^ΔC_3_ (150–180 μm), ^M^ΔC_2_ (180–210 μm), and ^H^ΔC_1_ (210–240 μm) measured from the left boundary of the EID reservoir of the device. Each region measures roughly one-third of the microchannel length, L_OS_. (**C**) Concentration profile from the source to the sink reservoir of the μOS device using a 20 kDa FITC–dextran tracer for experimental measurement (circles) alongside computational simulations (solid line). (**D**) The linear region between the vertical red lines corresponds to the concentration profile within the L_OS_ segment lengths of the microchannel array.

The concentration profiles generated within the reservoir denoted as the EID chamber (sink) and the BL chamber (source), as well as the horizontal microarray of the µOS device were modeled computationally (solid line) and validated experimentally (black circles), as shown in Figure 4. In this model, the µOS is continually flushed with maximum reagent, C = C_o_, on the source reservoir (right, color red) and cell media, C = 0, on the other (left, color blue). The steady-state concentration distributions shown in Figure 4B are normalized to the inlet concentration, C_o_, to highlight distinct regions of concentration within the µOS system. A steady-state concentration gradient is developed along each of the 90-µm-lengths of the OS microarray (L_OS_) after several minutes, as seen from the linear concentration distribution within dextran-loaded µOS devices in Figure 4D.

As Fick’s law dictates a linear steady-state concentration gradient for one-dimensional flows, one unique benefit of the µOS is the ability to examine RNB behavior within different concentration ranges of the same gradient field. This is highly advantageous because numerous groups, including our own [26,36,41,42], have illustrated the importance of concentration and concentration gradient, separately, in the motile behavior of different types of cells. The linear concentration gradient within the microarray has been divided into three separate concentration regions along the microchannel, which are grossly denoted as High gradient, ^H^ΔC_1_, Medium gradient, ^M^ΔC_2_, and Low gradient, ^L^ΔC_3_, as shown in Figure 4D. These ranges were chosen to depict changes in concentration by approximate orders of magnitude. In Figure 4D, the average concentration region ^H^ΔC_1_ is denoted between 210 and 240 µm of the microchannel from the EID, or source chamber, and it has a normalized concentration value, (C/C_o_)_1_, between 0.65 and 0.80. The concentration region ^M^ΔC_2_ spans the microchannel length 180 to 210 µm from the EID chamber with a normalized concentration, (C/C_o_)_2_, between 0.40 and 0.65. The concentration region ^L^ΔC_3_ is located 150 to 180 µm from the EID chamber with a normalized concentration value, (C/C_o_)_3_, from 0.25 to 0.40. These data highlight a 99.87% similarity between the computationally derived spatial concentrations and experimental measurements of fluorescent dextran within the system.

**Figure 5:**
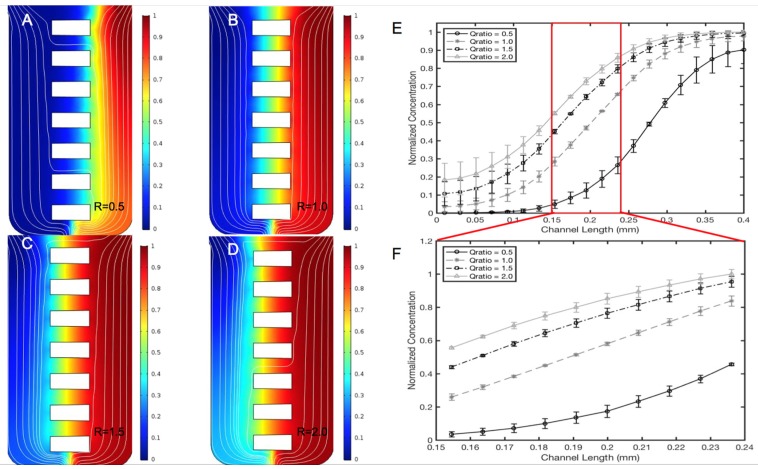
Concentration profiles of targeted fibroblast growth factor (FGF) ligand are generated within the µOS using different volume flow rate ratios of the left and right inlet ports, R = Q_R_/Q_L_. (**A**) The concentration profile within the μOS system when R = 0.5 is used, which is generated when the volume flow rate through the left, or sink port, Q_L,_ is twice the volume flow rate through the right, or source port, Q_R_. (**B**) The concentration profile within the μOS system when the volume flow rate ratio, R, is equal to 1.0. (**C**) The concentration profile within the μOS system when R is equal to 1.5 and (**D**) when R is equal to 2.0. (**E**) Concentration profile along horizontal positions of the μOS device for varying volume flow rate ratios, 0.5 ≤ R ≤ 3.0. The region between the vertical lines denotes the microarray. (**F**) The inset demonstrates the concentration profiles along the microarray for varying volume flow rate ratios, 0.5 ≤ R ≤ 3.0.

Different ratios of inlet volume flow rates on the left port, Q_L_, and right port, Q_R_, were used to modify the length of distinct concentration ranges within the same concentration gradient, which are denoted as ^H^ΔC_1_, ^M^ΔC_2_ and ^L^ΔC_3_ in Figure 4B. The images in Figure 5 illustrate that the volume flow rate ratios R = (Q_R_/Q_L_) between 0.5 and 3.0 produced different regions of concentration fields at different spatial lengths of the microarray. Figure 5B shows that a volume flow rate ratio of R = (Q_R_/Q_L_) = 1.0 is optimal for equal lengths of average concentration regions along all µOS channel arrays. Figure 5E denotes the different concentration distributions along the μOS produced by using a range of volume flow rate ratios, R. The distributions are seen to follow similar patterns in every instance. Figure 5F quantitatively illustrates that a volume flow rate ratio of R = 1 produces linear concentration gradients in the microarray, whereas volume flow rate ratios greater than one (R > 1) and less than one (R < 1) produce non-linear concentration gradients within the array of the µOS. Linear regression in this region produces an R^2^ value of 0.9999 for the R = 1 curve and R^2^ values between 0.94 and 0.99 for all other R values, further supporting this linear gradient assertion. Microenvironments produced with volume flow rate ratios not equal to one are consistent with FGF transport via convective diffusion, where the imbalance of Q_L_ and Q_R_ at the inlet ports generates a small bulk velocity along the channels of the microarray [56,58,70]. These data illustrate the tunability of the µOS system to measure RNB responses to local concentration fields on the order of tens of microns within the same gradient field. This unique advantage enables study of the effects of concentration and concentration gradient on RNB migration, independently as well as in concert. This ability is highly significant for RNBs, and other progenitors, as they are able to migrate varied anatomical distances during development and/or regeneration.

### 3.3. Collective Migration of RNB Clusters

Primary RNBs were loaded into the EID reservoir of µOS devices whose inner surfaces were coated with ConA, a priori, to facilitate adhesion for 1–2 h [37]. Then, FGF solution was continuously flushed into the BL chamber of the µOS (source reservoir, right) and allowed to transport to the EID chamber (sink reservoir, left), which was similarly flushed with solutions of media only. All experiments maintained continuous flushing of the system reservoirs using an equal volume flow ratio of R = (Q_L_/Q_R_) = 1. Volume flow rates Q_L_ and Q_R_ of 1 µL/min, each, were used to load the system for each test within minimal shear stress [62]. A linear FGF concentration gradient was produced along the L_OS_, as per Fick’s Law, to stimulate RNB chemotaxis. 

The majority of RNBs in the µOS microarray responded to FGF signaling fields as cohesive cell clusters of varying mean size, as shown in Table 2. Individual cells comprised 14% of the RNBs within the microarray but were not observed to migrate appreciably within the interstitial spaces of the L_OS_. This result is consistent with our previous studies [36,40] demonstrating that individual RNB did not chemotax more than 1–2 cell diameters toward FGF signaling. Motile RNB clusters were observed to cross the L_OS_ in two main configurations: (1) small clusters of 3–5 cells (65% of total motile population) and (2) large clusters of greater than 5 cells (21% of total motile population). Smaller clusters of RNBs were seen to migrate along the L_OS_ without disaggregation into singleton cells. These RNB clusters exhibited collective migration along the linear concentration gradient without significant differences in net displacement within each of the three concentration regions shown, ^H^ΔC_1_, ^M^ΔC_2_, and ^L^ΔC_3_. Table 2 lists the average total path length, Lp, net displacement, D_N_, and directionality, D, describing the behavior of RNBs observed in control conditions (media only) and FGF fields. In controls (media only), single cells and small clusters displayed an average total movement of less than 1 cell diameter (3–4 µm) along the L_os_ of the microarray with very low directionality, DR < 0.31. By contrast, small RNB clusters responded to FGF signaling with an average total path length of L_T_ = 17.7 ± 1.83 and an average net displacement of D_N_ = 10.1 ± 0.99. In addition, small clusters exhibited a high directionality in the horizontal plane with an average value of DR = 0.72 ± 0.15 in response to FGF. These data suggest that the migration of smaller RNB clusters is more dependent on the concentration gradient field than the absolute concentration threshold, as supported by numerous studies [55,71,72] identifying gradient effects as dominant forces in chemotaxis. Further, previous study from our group used microdevices of much larger dimensions (>100 µm) to illustrate a similar preferred cluster migration of retinal progenitors in the 3–5 cell range [36]. These data corroborate previous work from our group [36,37,40,42] illustrating that RNBs and retinal progenitors migrate small distances, collectively, in response to chemotactic fields. Numerous groups [73,74,75,76] have similarly demonstrated that neural progenitors migrate much smaller distances, in vitro, that other cell types, such as fibroblasts [77,78].

### 3.4. Disaggregation of Large RNB Clusters

**Figure 6:**
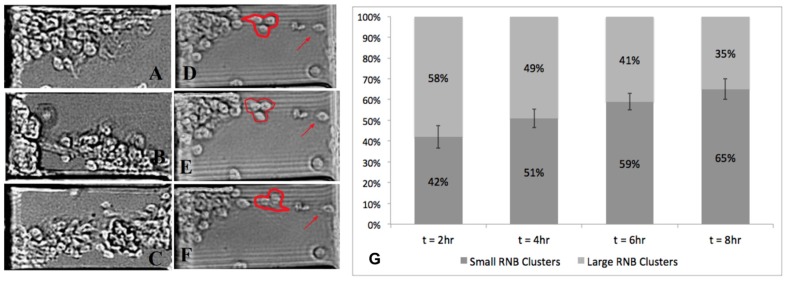
Migration of large Drosophila-derived RNB clusters within defined FGF concentration fields and gradients generated within μOS device. (**A**,**B**) Bright field images of intact and motile large RNB clusters within the microarray length of the device. (**C**) A representative, large RNB cluster that migrates en masse along the L_OS_ segment of the μOS system. (**D**–**F**) A typical large cluster exhibiting disaggregation, where smaller RNB clusters are seen to disassociate within the microarray and achieve longer motile distances than the original larger RNB. Arrows point to individual RNBs within L_OS_ segments that remain non-motile alongside motile clusters. (**G**) Ratio of small and large motile RNB clusters in the μOS device over the experimental time scale. Scale bar is 35 μm.

Large RNB clusters of more than 5 cells were observed to migrate across the microarray via three patterns, as shown in Figure 6. First, groups of large clusters were seen to migrate collectively across the L_OS_ in conventional chemotaxis, as seen in Figure 6A,B. Second, a portion of larger clusters migrated en masse, i.e., traversed the entire microchannel length from sink to source reservoirs without disaggregating into smaller clusters, as shown in Figure 6C. These large clusters exhibited net displacements equal to the length of the microarray, D_N_ = L_OS_, and L_T_ could not be determined (i.e., clusters grew as the cells migrated en masse). Third, a portion of large clusters exhibited disaggregation during migration within the L_OS_, where RNBs detached from large clusters to form smaller collectives, as shown in Figure 6D–F.

Then, we examined the disaggregation of large RNB clusters within the linear concentration gradient of the L_OS_ over the 8-h experimental time period. Figure 6G illustrates the pattern of motile disaggregation from larger RNB clusters over time within the same gradient field. At t = 2 h, 42% ± 11% of motile clusters were comprised of 3–5 cells (i.e., small RNB clusters), and the remaining 58% consisted of more than 6 cells (i.e., large RNB clusters). However, by t = 4 h, larger motile clusters had begun to disaggregate such that 51% ± 9% of motile clusters were comprised of 5 or less RNBs. Similarly, at t = 6 h, 59% ± 8% of motile clusters were small RNBs, and by t = 8 h, a larger 65% ± 10% of the motile clusters migrated within collectives of 3–5 cells. Importantly, Z stack imaging verified that the maximum height of large RNB clusters was less than the 10-µm height of the µOS array, indicating that RNB disaggregation was not driven by the confinement effects of the channel walls. Further, previous studies by our group used microdevices of much larger dimensions (>100 µm) to illustrate similarly preferred cluster migration of retinal progenitors in the 3–5 cell range [36]. This so-called motile disaggregation of larger RNB clusters occurred predominantly in the low concentration region, ^L^ΔC_3_, and medium concentration region, ^M^ΔC_2_. None of the clusters (0%) exhibited disaggregation in the higher concentration range, ^H^ΔC_1._ However, we note that these regions are also tightly correlated to the geometry of µOS system, where cells enter the L_OS_ from the EID source reservoir.

This cluster RNB behavior points to the importance of ligand concentration on the cell–cell cohesion needed for collective RNB chemotaxis. Soluble factors are well-known to play significant roles in the function, maintenance, and response of stem-like cells during development [79], and changes in collective stem cell responses induced by extracellular fields are critical to the advancement of regenerative neural repair [80,81]. Recent projects have implicated molecules such as cadherins, tight junctions, and pannexins in the cell-to-cell adhesions that facilitate the collective response of RNBs [82]. Therefore, these interactions become critical targets for regenerative medicine using stem-like cells in retina and elsewhere in the nervous system. Our current data of the µOS suggest that changes in the absolute concentration of targeted ligands, in this case FGF, can impact cell–cell cohesion of collective motile RNBs. Additional investigation is needed to elucidate the role of cell-to-cell adhesions that drive RNB collective migration in preferential sizes of 3–5 cells in response to extracellular chemical stimuli, as well as the disaggregation of large RNBs observed. Future study will exploit the robust genetic strength of the Drosophila model to evaluate the roles of FGF receptor expression and distribution on motile cells in tandem with pannexin 1 and/or E-cadherin [83], which are the primary cell adhesion molecules for this model organism.

## 4. Conclusions

This project introduced a new μOS microfluidic system that recapitulates the geometric constraints of the developing optic stalk in Drosophila. The μOS device enabled study of the collective motile behavior of primary RNBs in response to exogenous concentration fields of FGF, which is a prominent growth factor in is retinal development. The system establishes distinct concentration regions within the same gradient field to enable the study of RNB collective, chemotactic behavior in response to both concentration and gradient. Experimental data illustrated the influence of exogenous concentration on the cell–cell cohesion needed for the collective chemotaxis of primary RNB cells. Future study will utilize the μOS system to examine the mechanistic effects of extracellular concentration fields on the cell-to-cell adhesions needed for the collective migration of neural stem-like cells.

## Figures and Tables

**Table 1:** Critical dimensions of developing optic stalk within the visual system of Drosophila Melanogaster.

Key Features of μOS	Dimensions of μOS (μm)	Key Features of Developing Eye	Dimensions of Developing Eye (μm)
Length of Optic Stalk (L_OS_)	90 ± 5	Diameter of Eye Imaginal Disc (D_EID_)	500 ± 23 [9,54]
Characteristic EID width of Optic Stalk (W_OS1_)	37 ± 3	Diameter of Brain Lobe (D_BL_)	800 ± 14 [9,54]
Characteristic BL width of Optic Stalk (W_OS2_)	35 ± 3	Length of Optic Stalk (L_OS_)	90 ± 2 [9,54]

**Table 2:** Migration and directionality of small RNB clusters in control conditions (Schneider’s medium, only) and in response to targeted FGF concentration profiles within a μOS device over the 8-h experimental time period.

Type	Definition	Percentage of RNBs (%)	Average Total Path Length, L_T_ (μm)	Average Net Displacement, D_N_ (μm)	Directionality, DR
Single Cells	1–2 cells	16%	2.67 ± 0.58	0.67 ± 0.58	0
Small Clusters	3–5 cells	65%	17.7 ± 1.83	10.1 ± 0.99	0.72 ± 0.15
Large Clusters	>5 cells	21%	14.2 ± 2.94	4.44 ± 2.01	0.79 ± 0.22
